# Supplementary material for: A transfer-aware, deployment-oriented evaluation framework for NetFlow-based intrusion detection systems (TAN-IDS)
Source: PLoS One. 2026 Apr 8;21(4):e0346801. doi: 10.1371/journal.pone.0346801 (PMC13061228; doi:10.1371/journal.pone.0346801)
Supplement: Attachment — Submitted filename: Response_to_Reviewers.docx [file pone.0346801.s001.docx]

**Response to Reviewers**

We sincerely thank the Academic Editor and the reviewers for their constructive and insightful comments on our manuscript entitled **“A transfer-aware, deployment-oriented evaluation framework for NetFlow-based intrusion detection systems (TAN-IDS)”**.

The manuscript has been carefully revised to address all comments raised during the review process. The revision improves the clarity, structure, and presentation of the work. In particular, the following changes have been made:

- clarification of the concept of *transfer-aware evaluation*
- improved explanation of the **8-dimensional NetFlow feature representation** and its deployment-oriented design rationale
- reduction of redundancy in the Introduction
- reorganization of figures to improve readability
- updates to the Related Work section with recent IDS literature
- additional discussion of limitations and external validity in the Threats to Validity section

All modifications are marked in the revised manuscript with tracked changes.
Detailed responses to each reviewer comment are provided below.

1. **Response to Reviewer #1**

**Comment 1**

Sections 1.5–1.7 appear repetitive and could be shortened.

**Response**

Thank you for this helpful suggestion. The Introduction has been revised to reduce redundancy and improve conciseness. Several paragraphs in Sections 1.5–1.7 were condensed and partially reorganized to avoid repetition while preserving the key motivation and contributions of the work. Overall, the Introduction has been shortened and repetitive explanations were removed.

**Location in manuscript**

Introduction section (pages 3–5, lines 89–149).

**Comment 2**

While the 8-dimensional representation enables excellent interoperability, it may risk stripping away nuanced data required to detect highly complex or novel attacks.

**Response**

We thank the reviewer for this insightful observation. We agree that the use of a compact 8-dimensional NetFlow representation introduces a trade-off between feature expressiveness and cross-dataset interoperability.

To clarify this limitation, we expanded the discussion in the **Threats to Validity (Construct Validity)** subsection. The revised text explicitly acknowledges that richer feature sets may capture more detailed attack semantics, including application-layer behaviors. However, the compact NetFlow interface adopted in TAN-IDS represents a deliberate **deployment-oriented design choice**, prioritizing cross-dataset comparability and reproducible evaluation across heterogeneous network environments.

This clarification emphasizes that the goal of TAN-IDS is not to maximize detection performance through richer feature representations, but to enable **consistent cross-domain evaluation under realistic flow-monitoring constraints**.

**Location in manuscript**

Section *Threats to Validity – Construct Validity*, lines 939–946.

**Comment 3**

The Related Work section should include additional recent literature.

**Response**

Thank you for the helpful recommendation. We reviewed the suggested publications and incorporated them into the Related Work section to strengthen the discussion of recent advances in machine learning–based intrusion detection.

The suggested works have been added as references **[57–59]**, and they are now cited and discussed to provide additional context on modern IDS detection techniques and evaluation approaches.

**Location in manuscript**

Section 2 – Related Work (references [57–59], lines 203–213 and 299–305).

2. **Response to Reviewer #2**

**Comment 1**

The definition of “transfer-aware” is overly vague.

**Response**

We appreciate this important comment. In the revised manuscript, we clarified the definition of *transfer-aware evaluation*. In the context of this study, transfer-aware refers to evaluation protocols that explicitly consider **domain shift between training and deployment environments**.

Specifically, the framework organizes several evaluation scenarios that reflect realistic deployment conditions, including:

- cross-dataset transfer (training and testing on different datasets),
- mixed-domain training,
- limited-label target-domain fine-tuning.

This clarification distinguishes transfer-aware evaluation from conventional single-dataset experimental protocols.

**Location in manuscript**

Section *1.4 Beyond accuracy: transfer-aware and explainable evaluation*, lines 82–85.

**Comment 2**

Using only two datasets results in insufficient external validity.

**Response**

We thank the reviewer for raising this important point. In the current study, **UNSW-NB15** and **NF-CSE-CIC-IDS2018** were selected because they represent two widely used IDS benchmarks with substantially different traffic generation methodologies, attack distributions, and feature characteristics.

These datasets allow us to study domain shift under controlled conditions while maintaining a consistent NetFlow-level feature interface.

Importantly, **TAN-IDS is designed as an evaluation framework rather than a dataset-specific model**. The framework operates on a unified NetFlow feature representation and can be applied to additional flow-based IDS datasets without modification. To clarify this scope, we expanded the discussion of dataset limitations and external validity in the revised manuscript.

Future work will extend the evaluation to additional benchmarks to further assess cross-domain robustness across a broader range of network environments.

**Location in manuscript**

Section *Threats to Validity – External Validity*, lines 972–977.

**Comment 3**

Fine-tuning utilized only 5% of the data, with no ablation experiments conducted.

**Response**

Thank you for this insightful comment. The 5% labeled target-domain proportion was selected to simulate a realistic **low-label adaptation scenario** commonly encountered in operational IDS deployments, where only limited annotated traffic is available in a newly monitored environment.

In the revised manuscript, we clarified this design choice in Section 1.7. The objective of this scenario is to illustrate how the TAN-IDS framework evaluates performance recovery under limited-label transfer conditions rather than to optimize adaptation strategies.

Preliminary observations indicate that even small labeled subsets can provide meaningful improvements in cross-domain detection performance, supporting the feasibility of lightweight adaptation in practice.

Future work will further investigate systematic ablation of fine-tuning ratios and alternative adaptation strategies across multiple datasets.

**Location in manuscript**

Section *1.7 Evaluation scenarios and training strategies*, lines 140–149.

**Comment 4**

Why choose 8 dimensions?

**Response**

We thank the reviewer for this helpful observation. The 8-dimensional NetFlow representation was selected as a **compact and deployment-oriented feature interface** that can be consistently extracted across heterogeneous flow-based IDS datasets.

These features correspond to core flow-level statistics commonly supported by NetFlow exporters and monitoring infrastructures. Restricting the representation to this minimal and interoperable feature set helps avoid dataset-specific feature dependencies and enables fair cross-dataset comparison.

This design reflects a **deployment-oriented minimal interface**, rather than an attempt to maximize detection performance using richer dataset-specific features.

**Location in manuscript**

Section *3.2 Unified NetFlow feature space*, lines 356–361.

**Comment 5**

Figures are too dense. Merge Figures 3–7.

**Response**

We appreciate this helpful suggestion. To improve readability, the plots were reorganized into figures with multiple subpanels. Related results are now grouped into consolidated figures showing macro-F1 and attack recall side by side for each evaluation scenario.

This restructuring reduces visual clutter and improves comparison across scenarios.

**Location in manuscript**

Section 5 – Experimental Results (Figures 3–4).

**Comment 6**

Long sentences and dense paragraphs reduce readability.

**Response**

We thank the reviewer for this helpful suggestion. The manuscript was carefully edited to improve clarity and readability. Several long sentences were revised and shortened, and minor stylistic edits were applied to reduce sentence complexity and improve the flow of the text.

These revisions improve the overall readability of the manuscript.
